# Pericardial Tamponade: An Uncommon Clinical Presentation in cANCA Related Vasculitis and Glomerulonephritis in Association with Very High Titres of ANA

**DOI:** 10.1155/2019/4983139

**Published:** 2019-06-13

**Authors:** Amaresh Vanga, Amna Z. Rana, Jolanta Kowalewska, Harlan Rust

**Affiliations:** ^1^Nephrology, Eastern Virginia Medical School, 855 W Brambelton Ave, Norfolk, VA 23510, USA; ^2^Eastern Virginia Medical School, Norfolk, VA, USA; ^3^Pathology, Eastern Virginia Medical School, 855 W Brambelton Ave, Norfolk, VA 23510, USA

## Abstract

ANCA (anti-neutrophil cytoplasmic antibody) vasculitides are systemic autoimmune diseases in which anti-neutrophilic cytoplasmic antibodies activate primed neutrophils, thereby generating an inflammatory cascade resulting in the damage of small sized blood vessels in various organs of the body, including the heart. Pleuropericardial involvement is underrecognized as a complication of ANCA vasculitis and is highlighted in this case report of a 51-year-old male who presented with an initial symptomatic presentation of pleuropericardial effusion progressing to pericardial tamponade in the setting of a later renal biopsy proven pauci-immune crescentic glomerulonephritis with high ANA titres along with positive cANCA (cytoplasmic ANCA) and PR3 (proteinase 3) antibodies. He was found to have acute renal failure which progressively got better with cyclophosphamide.

## 1. Introduction

ANCA (anti-neutrophil cytoplasmic autoantibody) vasculitis consists of multiorgan related small vessel pathology due to the generation of autoantibodies that activate neutrophils and generate an inflammatory cascade. There are two major groups of ANCA vasculitis based on the target antigens: proteinase-3 (PR-3) and myeloperoxidase (MPO) [1]. PR-3 is associated with cytoplasmic neutrophilic staining (c-ANCA) and MPO is associated with perinuclear neutrophilic staining (p-ANCA). PR-3+ c-ANCA vasculitis generally presents with granulomatosis with polyangiitis (GPA), formerly known as Wegener's vasculitis, whereas p-ANCA has traditionally been related to microscopic polyangiitis (MPA) and eosinophilic granulomatosis with polyangiitis (EGPA), formerly known as Churg-Strauss syndrome [2]. The various clinical and pathologic manifestations of ANCA vasculitis include glomerulonephritis, respiratory complications including interstitial lung disease and upper respiratory tract ulcer formation, and dermatologic pathology such as palpable purpura and urticarial vasculitis [1, 2]. In this case report of a 51-year-old male with ANCA positive serology, we discuss the rarely associated ANCA related pleural and pericardial involvement with progression to pericardial effusion.

## 2. Case Presentation

A 51-year-old white male, with a past medical history of hypertension and hypothyroidism, presented with a 12-day history of shortness of breath, cough, and fever with new onset lower extremity swelling, orthopnea, paroxysmal nocturnal dyspnea, and dyspnea on exertion. He was seen by his primary care physician approximately one week ago and started on azithromycin, but did not improve. He had also been taking 5 mg of motrin and had used 30 tablets of motrin in the past week. His other medications included amlodipine 10 mg once daily and levothyroxine 50 mcg once daily. He was afebrile on initial presentation. Physical exam was pertinent for rales auscultated in the left lower lung base. EKG only pertinent for sinus tachycardia with no ST segment changes. His initial labs were pertinent for findings of new onset acute renal failure with a creatinine (Cr) of 3.4 mg/dL, microscopic hematuria and proteinuria on urinalysis, with urine protein: creatinine ratio of 1.34 g/gCr. He had appreciable leukocytosis with white blood cell count of approximately 20k. CT chest revealed small to moderate sized bilateral pleural effusions and moderate to large sized pericardial effusion. He was admitted to the inpatient service. Over the next 48 hours, he developed worsening shortness of breath, hypoxemia, and pericardial tamponade with echocardiogram (ECHO) revealing a worsening large circumferential pericardial tamponade in comparison to an ECHO done the previous day. ECHO also noted paradoxical septal motion during cardiac cycles with diastolic collapse of the right ventricle and right atrium. Pericardiocentesis was performed and approximately 500 mL of serosanguinous fluid was drained from the pericardial space with noted improvement in the patient's blood pressure and heart rate (see Tables 2 and 3 for pericardial studies).

**Figure 1:**
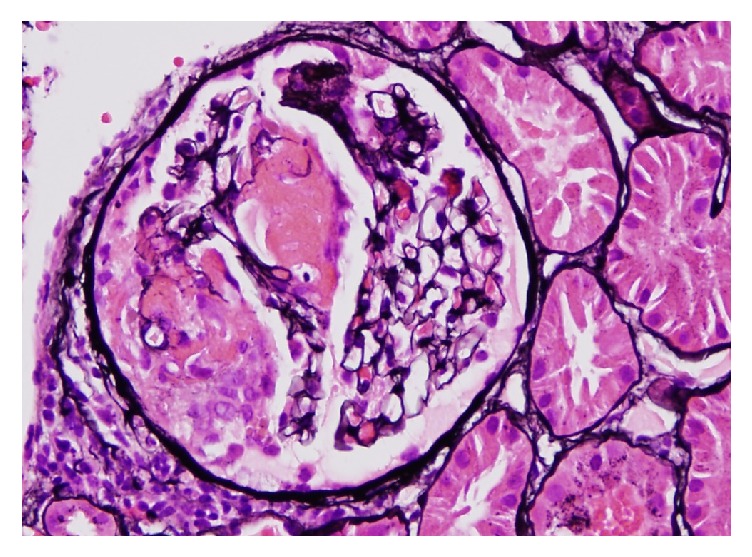
Glomerulus with segmental necrosis characterized by rupture of capillary wall and accumulation of fibrin and a segmental cellular crescent (by periodic acid-methenamine silver (PAM) with hematoxylin and eosin staining, original magnification 200x).

**Figure 2:**
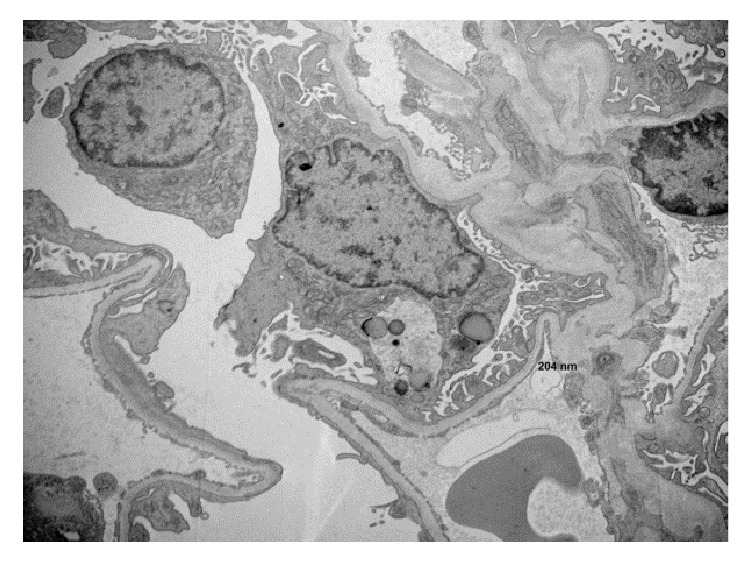
No abnormal deposits found on sampled glomerulus.

Further workup revealed positive autoantibodies for c-ANCA (1:160), ANA (1:1280) and PR-3 (>100). Anti-SSA (Sjogren's Syndrome–A), anti-SSB (Sjogren's Syndrome–B), anti-dsDNA (double stranded-DNA), anti-Smith, anti-RNP (ribonucleoproteins), and anti-GBM (glomerular basement membrane) were reported negative (Table 1). This prompted a renal biopsy which revealed fibrinoid necrosis, capillary wall rupture, fibrin formation, and cellular crescents consistent with rapidly progressive crescentic glomerulonephritis (Figure 1). Examination of provided electron microscopy images showed 2 glomeruli which revealed no evidence of immune type electron dense deposits in the glomeruli, tubular basement membranes, or the interstitium (Figure 2).

Normal findings on electron microscopy were thought to be related to sampling error. Immunofluorescence revealed pauci-immune pattern. Based on the renal biopsy, he was diagnosed with PR3-ANCA related pauci-immune glomerulonephritis with extra renal manifestation of pleuropericardial disease progressing to pericardial tamponade. Patient then received pulse steroids and a dose of rituximab. BUN and Cr were 91 mg/dL and 3.2 mg/dL after getting this treatment. He was then discharged after one week of inpatient stay with plans to slowly taper steroids and to continue with weekly rituximab injections.

However, BUN/Cr worsened over the course of the next week to 176 mg/dL and 8.6 mg/dL, and thus he was concluded as rituximab failure and started on cyclophosphamide and plasmapheresis in the inpatient setting.

**Figure 3:**
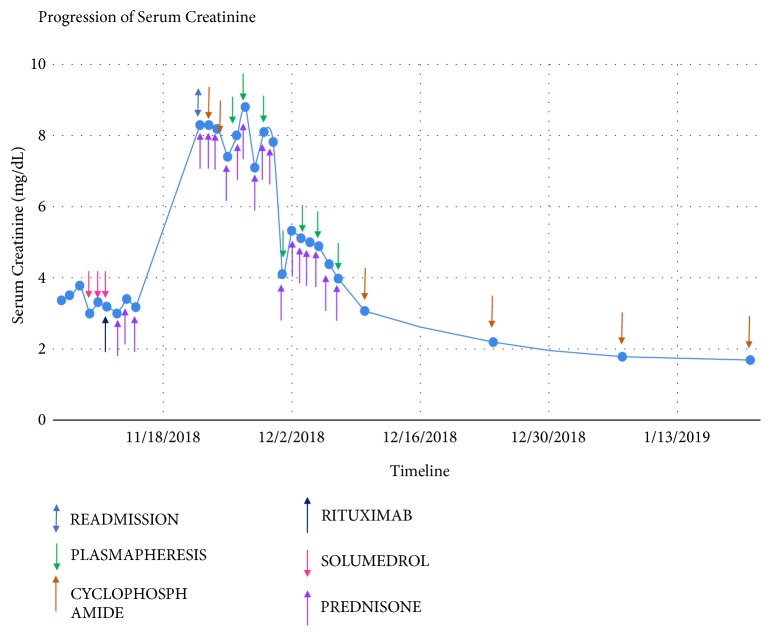
Creatinine response to treatment regimen.

An ECHO done on readmission showed a small fibrin filled pericardial effusion which required no clinical intervention. Chest X-ray (CXR) on readmission showed stable opacity consistent with prior pleural effusion. Pericardial effusion remained stable in size and patient remained asymptomatic when ECHO was repeated 10 days after readmission. After 7 sessions of plasmapheresis and treatment with 1200 mg IV cyclophosphamide, his creatinine stabilized at 1.7 mg/dL (Figure 3).

## 3. Discussion

The underlying pathophysiology of ANCA vasculitis is thought to be secondary to the formation of antibodies against key neutrophil antigens which are typically sequestered within the neutrophil and have migrated to the cell membrane of the neutrophil. This migration of typically sequestered antigens is thought to be secondary to cytokine release due to previous or concurrent inflammatory insult and is referred to as “priming” of neutrophils. Antibodies, such as anti-PR3 or anti-MPO, thus activate the neutrophil via interaction with their respective antigens and cause a respiratory burst with degranulation and release of complement activating factors with proinflammatory enzymes. This autoimmune process goes on to affect small-medium sized vessels with organ selectivity depending on the type of ANCA process [1]. Various case reports in the past have reported an association between ANCA vasculitides and pleuropericardial involvement [3–7]. However, cardiac involvement is more common in p-ANCA associated vasculitis including EGPA and it is reported that 13-47% of patients with EGPA have been found to have a myocardial, endocardial, or pericardial lesion with another source reporting 8-32% incidence of pericardial involvement in patients with EGPA [8–10]. Nonetheless, c-ANCA vasculitides have also been associated with cardiac involvement with a reported 19% incidence of pericardial involvement in GPA patients [9].

In this case report, the patient presented with an initial predominant presentation of pericardial tamponade and was then discovered to have renal biopsy proven PR3 pauci-immune glomerulonephritis. Pericardial effusions can be secondary to a wide range of etiologies, including idiopathic, malignancy, infectious, and systemic conditions such as hypothyroidism and autoimmune disorders [11]. In this case, pericardiocentesis yielded an exudative effusion, per Light's criteria, with a fluid protein: serum protein ratio of .75. Furthermore, cytology was pertinent for no malignant cells but indicated acute inflammation. Pericardial fluid gram stain and culture were negative. Blood, respiratory, and urine cultures were also negative. Workup lowered suspicion for infectious or malignant etiologies. Normal complement levels with an autoimmune workup negative for all other antibodies, including dsDNA, SS-A, SS-B, anti-Jo 1, and anti-Scl, lowered the suspicion for lupus or other autoimmune pathologies. Hence, the remarkable ANA titres, positive PR-3, and cANCA antibodies with biopsy proven pauci-immune crescentic glomerulonephritis created the case for vasculitis associated pericardial involvement with progression to pericardial tamponade. Of note, a biopsy of the heart could have further elucidated the role of vasculitis induced pericardial effusion. However, it was felt to be unnecessary given the timeline, presentation, and associated positive findings in laboratory workup and renal biopsy.

This case report is significant as it highlights the importance of considering a pericardial effusion as the initial presenting sign of a vasculitis. It certainly underscores the point that ANCA vasculitides are systemic diseases which may go on to affect various organs in the body, aside from the pathognomonic presentations.

## Figures and Tables

**Table 1:** Laboratory workup.

Labs	Reference Values	Values
*White Blood Count*	*4.0 - 11.0 K/uL*	*20.7*
*ANA Screen by IFA*	*Negative*	*Positive*
*ANA Speckled*	*Negative Titer *	*> = 1:1280*
Anti-dsDNA	Negative	Negative
Anti-GBM	0-20 units	5
C3 Complement	83 - 177 mg/dL	162
C4 Complement	10 - 40 mg/dL	16
*Cytoplasmic ANCA*	*Neg: <1:20 titer*	*1:160*
*PR-3 Antibody EIA*	*0.0-3.5 U/mL*	*> 100*
MPO Antibody, EIA	0.0-9.0 U/mL	< 9.0
Perinuclear ANCA	Neg: <1:20 titer	<1:20 titer
*Urine Protein*	*Negative *	*100*
*Urine RBC*	*0-2/hpf*	*50-100*
*Urine WBC*	*0-2/hpf*	*20-50*
*Urine Protein: Creatinine*	*< .2*	*1.34 *

**Table 2:** Pericardial studies.

Pericardiocentesis	Results
*Pericardial LDH *	*1784 U/L*
Serum LDH	208 U/L
*Pericardial Protein *	*5 g/L*
Serum Protein	6.7 g/L
Culture and Gram Stain.	No bacterial growth noted. No anaerobes cultured. No acid fast bacteria seen at 200x magnification. No organisms seen on gram stain.
Cytology	Negative for malignancy.
	Marked acute inflammation present.
Pericardial Glucose	< 19 mg/dL
Pericardial Triglycerides	87 mg/dL
Serum Triglycerides	152 mg/dL
Pericardial Cholesterol	85 mg/dL
Serum Cholesterol	96 mg/dL

**Table 3:** Pericardial studies.

Appearance Fluid	Latest Ref Range: Clear	Bloody (A)
Condition Fluid	Latest Ref Range: No clot present	No clot present

WBC Fluid	Latest Units: /cumm	34,295

Neutrophils Fluid	Latest Units: %	90

Lymphocytes Fluid	Latest Units: %	1

Macrophages Fluid	Latest Units: %	9

LDH FLUID	Latest Units: U/L	

AMYLASE FLUID	Latest Units: U/L	56

SPECIFIC GRAVITY FLUID	Unknown	1.033

pH Fluid	Latest Units: pH	6.53
