# Corrigendum to “Pericardial Tamponade: An Uncommon Clinical Presentation in cANCA Related Vasculitis and Glomerulonephritis in Association with Very High Titres of ANA”

**DOI:** 10.1155/2019/6703652

**Published:** 2019-08-22

**Authors:** Amaresh Vanga, Amna Z. Rana, Jolanta Kowalewska, Harlan Rust

**Affiliations:** ^1^Department of Nephrology, Eastern Virginia Medical School, 855 W Brambleton Ave., Norfolk, VA 23510, USA; ^2^Eastern Virginia Medical School, Norfolk, VA, USA; ^3^Department of Pathology, Eastern Virginia Medical School, 855 W Brambleton Ave., Norfolk, VA 23510, USA

In the article titled “Pericardial Tamponade: An Uncommon Clinical Presentation in cANCA Related Vasculitis and Glomerulonephritis in Association with Very High Titres of ANA” [1], there were some errors as follows.

1. There was an error in the “Case Presentation” Section, where “5 mg of motrin” should be corrected to “5 tabs of Motrin at a time.”
2. There was an error in the “Abstract,” where the sentence “a 51-year-old male who presented with an initial symptomatic presentation of pleuropericardial effusion progressing to pericardial tamponade in the setting of a later renal biopsy proven pauci-immune crescentic glomerulonephritis with high ANA titres along with positive cANCA (cytoplasmic ANCA) and PR3 (proteinase 3) antibodies” should be corrected as follows: “a 51-year-old male who had an initial symptomatic presentation of pleuropericardial effusion that later progressed to pericardial tamponade in the setting of a renal biopsy proven pauci-immune crescentic glomerulonephritis. This patient also had high ANA titers along with positive cANCA (cytoplasmic ANCA) and PR3 (proteinase 3) antibodies.”
3. There was an error in the “Discussion” Section, where the sentence “In this case report, the patient presented with an initial predominant presentation of pericardial tamponade and was then discovered to have renal biopsy proven PR3 pauci-immune glomerulonephritis” should be corrected as follows.
4. “In this case report, the patient presented with an initial predominant clinical scenario of pericardial tamponade and was then discovered to have renal biopsy-proven PR3 pauci-immune glomerulonephritis.”
